# ‘We eat together; today she buys, tomorrow I will buy the food’: adolescent best friends’ food choices and dietary practices in Soweto, South Africa

**DOI:** 10.1017/S1368980012003254

**Published:** 2012-07-16

**Authors:** Carlijn GN Voorend, Shane A Norris, Paula L Griffiths, Modiehi H Sedibe, Marjan J Westerman, Colleen M Doak

**Affiliations:** 1 Faculty of Earth and Life Sciences, Department of Health Sciences, Vrije Universiteit Amsterdam, Amsterdam, the Netherlands; 2 Medical Research Council/Wits Developmental Pathways for Health Research Unit, Department of Paediatrics, Faculty of Health Sciences, University of the Witwatersrand, 7 York Road, Parktown, 2196 Johannesburg, South Africa; 3 School of Sports, Exercise and Health Sciences, Loughborough University, Loughborough, UK

**Keywords:** Adolescent behaviour, Peer group, Obesity, Eating, Qualitative research

## Abstract

**Objective:**

To explore if and how female adolescents engage in shared eating and joint food choices with best friends within the context of living in urban Soweto, South Africa.

**Design:**

A qualitative, exploratory, multiple case study was conducted using semi-structured duo interviews of best friend pairs to ascertain their eating patterns, friendship and social interactions around dietary habits.

**Setting:**

Participants were recruited from three high schools in the urban township of Soweto, South Africa.

**Subjects:**

Fifty-eight female adolescents (twenty-nine friend pairs) still in high school (mean age of 18 years) were enrolled.

**Results:**

Although overweight rates were high, no association between friends was found; neither did friends share dieting behaviours. Both at school and during visits to the shopping mall, foods were commonly shared and money pooled together by friends to make joint purchases. Some friends carefully planned expenditures together. Foods often bought at school were mostly unhealthy. Availability, price and quality were reported to affect choice of foods purchased at school. Preference shaped joint choices within the shopping mall environment.

**Conclusions:**

Food sharing practices should be investigated in other settings so as to identify specific behaviours and contexts for targeted and tailored obesity prevention interventions. School-based interventions focusing on price and portion size should be considered. In the Sowetan context, larger portions of healthy food may improve dietary intake of fruit and vegetables where friends are likely to share portions.

In Africa, as elsewhere, obesity rates are rising^(^ ^1^ ^)^. In South Africa, the overall prevalence of being overweight or obese is particularly high (57 %) among women^(^ ^2^ ^)^. Results from Cape Town show an even higher prevalence in historically disadvantaged townships, where 80 % of the females were found to be overweight or obese even though child undernutrition was also a major concern in the same community^(^ ^3^ ^–^ ^6^ ^)^. A disadvantaged urban South African township is Soweto, which comprises several townships positioned in the south-western part of the Johannesburg metropolis. It has one of South Africa's highest population densities^(^ ^7^ ^)^, with an estimated 1–1·5 million people^(^ ^8^ ^)^, and a prevalence of overweight in 17-year-old females of 27 %, which is higher than the national average^(^ ^2^ ^,^ ^9^ ^)^. Furthermore, high consumption of fast foods has been documented: on average females and males consume eight fast food items per week in Soweto^(^ ^9^ ^)^. The age of late adolescence and early adulthood is of particular relevance for females given the start of obesity at a young age^(^ ^2^ ^,^ ^10^ ^)^ and the high risk of overweight/obesity in adult females.

Research into adolescent food choice has focused on individual factors, including taste, familiarity/habit, health, dieting and satiety^(^ ^11^ ^,^ ^12^ ^)^. Multiple studies also indicate the influence of peers on food intake and food-related behaviours^(^ ^13^ ^–^ ^17^ ^)^. The mere presence of a friend while eating was shown to increase food intake in adolescents^(^ ^15^ ^)^ and friends were found to be important predictors of the subjective norm related to eating patterns^(^ ^18^ ^)^. Understanding the role friends play in the mutual shaping of the formation of identity and food consumption patterns may be helpful to identify strategies to effectively influence healthy adolescent food behaviour^(^ ^19^ ^)^. While peers exert an important influence on adolescent behaviour^(^ ^11^ ^,^ ^16^ ^,^ ^20^ ^)^, most prior research has focused on the influences in relation to intakes. No prior research has explored the process of food choices made by friend pairs in the urban African context.

Sharing with friends reinforces social bonds while potentially resulting in new consumption patterns for one or both of the friends. While most studies use the individual as the unit of analysis, research is needed to explore food choices and eating behaviours that are shared because food choices, especially in the adolescent age group, are often not made individually. Understanding the food-related behaviours of adolescents requires a model that includes cultural, social and biological/personal influence as described in the theory of triadic influence^(^ ^21^ ^–^ ^23^ ^)^. The theory has been successfully used in previous nutrition research^(^ ^24^ ^,^ ^25^ ^)^ including adolescent behaviour^(^ ^22^ ^,^ ^23^ ^)^. In the context of an obesogenic environment, such as in Soweto, the theory of triadic influence provides a framework for exploring the influence of the broader cultural environment as well as the social context of the school and home in relation to the food choices friends make together, while also taking into account the friends’ own individual characteristics. The present study aimed to explore if and how best friend pairs of female adolescents at the verge of adulthood engage in shared eating and joint food choices in the context of living in Soweto, South Africa.

## Methods

The present research uses a qualitative exploratory multiple case study approach^(^ ^26^ ^)^ and a duo-interviewing technique^(^ ^27^ ^)^. The study was carried out by a team of researchers with diverse academic and socio-cultural backgrounds.*

### Population and sampling

The target study population was grade 12 students (i.e. last year of high school). Four high schools identified by local researchers as ‘long standing’ in the community were chosen from different areas of Soweto. Of the four schools approached, one declined participation out of concerns related to student exams. The researchers visited each of the three participating schools to invite grade 12 girls to participate in the study and to be interviewed together with their best friend. A best friend was defined as ‘someone of your own age, you know very well, with whom you meet regularly (i.e. couple of times a week), you are engaged in activities, hang out and/or chill out with and you share emotional moments. This can be someone from the same neighbourhood and may not be from the same school’. The researchers provided informed consent forms for students to take home to their caregivers. Among all schools, thirty-two students returned the consent forms for themselves and for their best friend to their teacher. The written informed consent of all caregivers was confirmed by telephone (C.G.N.V.). All thirty-two pairs were then invited for an interview that took place at the Research Unit at Chris Hani Baragwanath Hospital. Two participant pairs were lost to follow-up, and one pair of students changed their mind and decided they did not want to participate. In total, fifteen, nine and five friend pairs were included for participation from the three schools.

### Interview method

Duo interviewing may improve the quality of information gathered and encourages in-depth discussion^(^ ^27^ ^)^. Participants may build upon each other's responses^(^ ^28^ ^)^ and point out divergent statements. Furthermore, the duo interview allows for an analysis that focuses on the answers of the pair (the duo), rather than individual answers, reflecting the normative behaviour of the two friends. In particular, through the process of giving an answer, agreeing or disagreeing with each other, the duo interview illustrates the decision process the friends make, as a unit. Other studies have successfully applied the duo-interviewing method in nutrition research^(^ ^25^ ^,^ ^28^ ^)^ although to our knowledge no studies have previously used this method to explore shared eating.

### Interview design

An interview topic guide was developed with a set of questions informed by aspects of the theory of triadic influence^(^ ^21^ ^,^ ^25^ ^)^ that were thought to be relevant to social bonding and food behaviours. The topic guide and formulated questions were piloted in four interviews. Questions were reformulated and new emerging concepts were probed through additional questions. For example, a number of participants mentioned pooling money together to purchase foods and this was identified as a new theme. Box 1 shows the revised main starting interview questions. In the pilot phase, multiple interviewers from different socio-cultural backgrounds were used. The interviewers were then evaluated based on quality and depth of information and level of participant comfort achieved. Based on these criteria, a local interviewer (M.H.S.) was appointed as the most successful interviewer. M.H.S. was able to conduct all interviews in a combination of local languages and was familiar with township culture and food items.

Box 1 Examples of interview questions

Friendship

So let's talk about your friendship, how did you meet?

What things do you do together?

Do you spend time together during school breaks?

Did you introduce any new foods to each other since you've known each other?

Food choices

Tell me about your day today, from when you got up what did you do?

Did you eat anything before going to school today? Why (not)?

What did you eat during break at school today? With who?

Did you buy it at school or bring it from home? Why? Probe.

How do you decide what to eat? Probe.

Do share lunch with friends? How? Probe.

When you get home, do you guys eat? What do you eat?

What foods do you enjoy when you go out? How? Probe.

Let's talk about Soweto, what are the most popular activities in Soweto, things that make it unique? Any food items that you would call ‘Soweto food’?

### Interview procedure

The twenty-nine duo interviews were conducted from June to July 2009, each lasting approximately 90 min. The trained interviewer (M.H.S.) began the interview by clarifying the goal of the study, explaining confidentiality processes and building a rapport with participants. The pairs were encouraged to talk freely and to discuss shared behaviours as well as individual differences. The interviewer probed for further understanding of the social interaction and negotiations related to food choices. Interviews were carried out in English, Zulu, Sesotho or combined languages, to enhance the participants’ comfort and the quality of information shared. An observer (C.G.N.V.) took notes and measured weight and height after the interviews to enable an understanding of the current nutritional status of the participants. The study was approved by the Human Research Ethics Medical Committee of the University of the Witwatersrand and agreement to undertake the project in Soweto schools was obtained from the Director of Education for Soweto.

### Analysis

All interviews were audio-recorded, transcribed verbatim and translated into English where necessary. The final transcribed interviews were checked for quality by M.H.S. and C.G.N.V., with translation checked by M.H.S. and a multilingual research assistant. Out of the twenty-nine interviews, eight transcripts were of insufficient quality for transcription and translation. Audio recordings of these interviews were used in the final stage to confirm and check for contrary or new information. Content analysis of twenty-one transcripts was undertaken in an integrated approach^(^ ^29^ ^)^ with the aim to explore and understand normative food choices. Using transcripts of five interviews, M.H.S. and C.G.N.V. confirmed and agreed initial codes on themes characterizing the friendship bond (e.g. origin, meaning, activities involved in together), the context of eating (e.g. home, school, mall) and individual and environmental factors influencing food choice (e.g. dieting, bringing a lunchbox, financial constraints). Afterwards, both researchers independently analysed the data, exploring if and how the pairs were involved in each other's food choices; examining similarities and differences within and between the duos. Relevant parts of transcriptions were coded and extracted using Microsoft Excel. Consistency of coding between C.G.N.V. and M.H.S. was checked by two other researchers (C.M.D., P.L.G.). The strength of the interpretations was critically discussed by the research team. Quantitative data (i.e. weight, height, age and school attended) were collected according to appropriate methods and analysed for all twenty-nine pairs of best friends.

## Results

Findings in the following sections are presented to tell a logical story – providing context of the friendships, explaining how friends are engaged in shared eating and which food choices are made in the two main social/cultural contexts where friends were involved in shared eating. Friends’ interaction, influences from the environment and caregivers, as well as personal characteristics that influence food choices that friends make together, are described. The last section focuses on individual overweight, and if and how dieting plays a role in the friendship.

### Context: friendship and food

Most of the friendships started at school or, as in the case of one pair, because they lived close to one another. Apart from three other pairs that were relatives, the friendships had existed for 1·5 to 12 years (average 4·6 years). Predominantly (i.e. twenty-four of the twenty-nine duos) the best friends were attending the same school as one another. Two participants brought their best friend who was attending university and three others brought a friend from a different high school. Students in some pairs were both part of the same bigger group of friends; in other cases they individually had their own separate group of friends.

Best friends often interacted in multiple contexts, such as spending time outside school at home or at the mall:

We like the same things so we do a lot of things together. (Pair 25)

A few pairs also went to parties or church together and some reported sleeping over at weekends at each others’ homes. In some instances, the friend pair partly or temporarily lived in the same house; for example, one of the cousin pairs but also a non-relative friend pair. A few examples were seen of friends who did not spend all breaks together at school and who only had occasional contact outside school. Sometimes, one or both individuals reported having one or only a few friends:

We don't attend the same class. On breaks we see each other, not always because I either go to the library or stay in class being busy reading. And sometimes at home because, I stay in Klipspruit and she stays in Diepkloof, so we have to travel so most of the time. … but I am always busy, I don't get much time, but the time we get we make use of it big time. (Pair 19)

In addition to doing things together, best friends were also a source of social support:

We understand each other, I can tell her about what is going on in my life, I am open to her, I tell her my problems and she gives me a solution. (Pair 1)

If I have a problem within myself I go to her and if she has a problem she comes to me. (Pair 15)

Listening and being understanding, keeping secrets, encouraging and advising were characteristics that participants reported to value in their friends. Friends also accepted and valued their differences:

She is not a friend who just agrees to everything. (Pair 20)

We kind of show each other light. We don't judge each other. (Pair 15)

Some friends included their shared love of food when they were asked about their friendship:

We eat the same food, same clothes we share the same style. (Pair 15)

Shared food consumption was reported by most friends, and occurred mainly in the school environment and during visits to the shopping mall. Other contexts, such as eating at a friend's home, when going on trips with friends, or while visiting other family members or hanging out with larger groups of friends in the neighbourhood, were less frequently mentioned.

### The school environment: food choices

Food for lunch and other breaks was often bought, and only a small proportion of the participants took a lunchbox to school regularly. Most respondents reported getting money from caregivers to spend on lunch, with amounts varying from R5 up to R30 per day (equivalent to approximately $US 0·64–3·84). Some chose not to bring a lunchbox because they preferred getting the money:

I don't like to bring lunch; if I take lunch they are going to give me R5·00 [laughs] my money would go down if I bring lunch. They would never allow me to take lunch and still give me R20, never. (Pair 20)

While many respondents described bringing a lunchbox from home as undesirable or embarrassing, some respondents did not have a choice:

My mom says I must carry the lunchbox … she does not understand, she just wants us to carry the lunchbox; I don't get any money. (Pair 4)

Multiple adolescents explained bringing a lunchbox as being related to financial constraints of their caregivers:

Well in the middle of the month, maybe when my mother doesn't have money then I would come with lunch. (Pair 24)

**Fig. 1:**
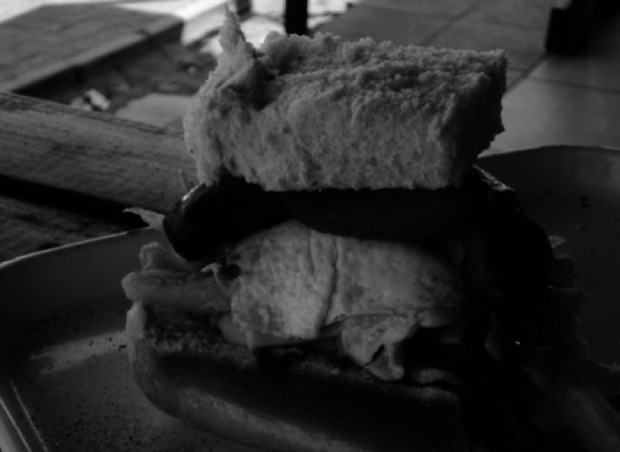
Example of a Sowetan ‘kota’, also known as a ‘quarter’ or ‘bunnychow’ (retrieved from Feeley *et al.* ^(^ ^9^ ^)^)

Tuck shops at or near schools were a popular source for food. The most popular item to buy at schools during this break was the relatively cheap, so-called ‘kota’; a quarter of a loaf of white bread with chips, meat, cheese, egg and/or sauce, as shown in Fig. 1.* Fruit juices and fatcakes (doughnut textured food item) were also regularly consumed. For smaller breaks small food items were popular to buy:

During first school break we buy kotas and cold drink, the next one crisps, the other one during study time we buy sweets and chocolate. (Pair 11)

The choice for particular food items was affected by different factors in the school environment such as the availability of food items, since at some schools food choices were limited:

But the one at school I eat it because it's there. (Pair 11)

Waiting time was for some no restriction, whereas others would rather choose something different to eat:

Though I like the kota, it takes a long time to prepare and my friends wait on the line for a long time, there's no line for cakes. (Pair 20)

Although the consumption of the kota was mentioned at all schools, buying and consuming healthy foods was reported by only some friend pairs, mostly from one particular school:

We buy oranges, apples … then I love avocados, we have plenty of options at school. (Pair 7)

At this school a hot lunch including rice, chicken and salad was also sometimes eaten as it was available for about the same price as a kota:

When we crave for take away plate we buy take away, and when we crave for kota we will buy kota, and when we feel like eating snacks we will buy fruits. (Pair 12)

The price of foods was mentioned to affect food choices; for example, one pair from another school did not buy fruits for this reason:

I bring mine from home, at school fruits are expensive. (Pair 11)

In addition, bad quality and taste of certain foods at school was reported multiple times as a reason to not buy these foods:

They [other friends] buy food from school, those taste horrible. (Pair 20)

I don't eat because I don't trust it, it's because I once ate mince then I had a runny stomach then I find out where I bought it, if the food was too much they would keep it on the fridge and warm them the next day like that until its tastes less. (Pair 11)

I don't like food from the tuck shop … sometimes they get burnt. (Pair 1)

### Sharing food and money at school

The majority of best friend pairs who attend the same school ate together during breaks and almost all of these pairs reported to share food regularly. Sometimes this happened within a bigger group of friends. When friends did not share with each other, mostly they shared with another (group of) friend(s). For example, a pair of cousins explained that they did not eat together because of their age difference. Few exceptions concerned cases where one of the friends spent a lot of time doing things alone while the friend ate with others:

Friend A: It's either I am in the library or in the class busy reading. Friend B: I buy food with my classmates and then we go and sit down and eat. (Pair 19)

The way food was shared differed between the pairs. Some took turns in buying food:

We eat together, we change turns; today she buys, and tomorrow I will buy the food. (Pair 11)

In other cases both friends reported bringing their own food to be shared:

I do buy a kota, we share the lunchbox and we share the kota. (Pair 11)

Sometimes food was shared to socially support friends who did not buy any:

If there is someone who did not buy or did not carry some food and then we share with them. (Pair 19)

We all share for that person or sometime you'll find that we give her money, maybe she'll like to buy snacks then we'll all contribute for her. (Pair 32)

Money was pooled together to make joint purchases by the majority of friends who shared their food, either between the two of them or in a larger group of friends:

We change turns; today she buys and tomorrow I will buy food. (Pair 11)

The amounts of money contributed by the friends were not necessarily equal:

Everyone says how much they have then we'd put it together. So everything we do we'd buy it together, so there isn't this thing that one has so much money, we buy together. (Pair 24)

Sometimes, joint purchases were made for practical reasons:

We have to combine it to one so that you don't have to stand in a long queue. (Pair 27)

As depicted earlier, money for food regularly came from home and some participants specifically planned to maximize the amount by not taking a lunchbox; while, for the same reasons, others decided to take a lunchbox to school:

I take food to school, I do get money, but I save my money. (Pair 25)

Furthermore, some friends planned together, for instance by choosing to use money that is given for food to buy other things:

We are saving for after exams, there are t-shirts we want to buy. … we ate today, we took out R5 every Friday; this Friday I won't eat, because I must save that R5. (Pair 29)

### Sharing food and money at the shopping mall

Time together with the best friend was often spent visiting a shopping mall. Like in the schools, friends also reported sharing their money to buy food here:

Everyone says how much they have then we'd put it together. So everything we do we'd buy it together. (Pair 24)

In general, mainly unhealthy foods were consumed at fast-food chains, which were very popular, and snacks were bought at small retailers or grocery stores. Food choices were generally made together, which was in some cases a compromise of alternating between the preferences of both friends:

We go there twice a month; the first week we'll buy KFC, then at month end we'll buy Wimpy, we compromise. (Pair 29)*

In other cases the choice was determined by whoever was paying:

Sometimes its Wimpy, we can't choose it's not our money. (Pair 11)

However, sometimes preferences differed and accordingly friends did not eat together:

Friend A: We argue about eating time because I'm on a diet and she wants to eat. Friend B: … when I want to eat it I go alone, I leave her behind. She doesn't eat it. She wants us to go to Pick ‘n Pay, and buy Lays, and buy some drinks and chocolate only. (Pair 25)†

### Overweight and dieting

More than half of the fifty-eight participants were overweight (Table 1), although less than a third (28 %) of the friend pairs were both overweight and no pairs were both obese. As most participants did not diet, dieting was generally not an issue in the friendship. However, in one case where both friends were overweight, one participant strongly disapproved of her friend dieting:

About her diet, well she knows I don't support it. She's on and off, like when she hears someone saying she's gained weight, she gets worried then she'll want to hold herself. (Pair 25)

**Table 1:** Anthropometric characteristics of the study participants: adolescent females (*n* 58), Soweto, South Africa

Measurement	Mean	sd	Min	Max
Age (years)	18·0	1·2	15·3	21·6
Height (cm)	157·4	5·6	139·9	169·5
Weight (kg)	64·6	14·3	43·7	109·1
BMI (kg/m^2^)	26·1	5·8	17·3	44·1
	*n*	%		
BMI international grades*				
Underweight (<−1 sd)	2	3·4		
Normal weight (−1 sd–1 sd)	26	44·8		
Overweight (1 sd–2 sd)	17	29·3		
Obesity class I (2 sd–3 sd)	10	17·2		
Obesity class II+III (≥3 sd)	3	5·2		

*For participants >19 years of age, cut-off points for classification were according to the WHO criteria for adults: underweight, BMI<18·5 kg/m^2^; normal weight, BMI = 18·5–24·9 kg/m^2^; overweight, BMI = 25·0–29·9 kg/m^2^; obesity class I, BMI = 30·0–34·9 kg/m^2^; obesity class II+III, BMI ≥ 35·0 kg/m^2^. For participants ≤19 years of age, classification of BMI category was adjusted for age according to the WHO growth reference data^(^ ^36^ ^)^.

Others also expressed opinions against dieting:

Have you ever dieted? I don't think I will be able to. (Pair 31)

No, like I love the way I look. (Pair 32)

Nevertheless, a few adolescents reported having dieted with their best friend or other friends:

At home if we find that we are fat, we try to slim. We have this thing in class that you would find us talking about going on a diet, we even went to the gym. (Pair 24)

Dieting was largely attributed to perceived body weight, although not necessarily related to obesity or overweight classification. Reasons for dieting were most often to look slim for a particular event, most often the matric dance:

Like right now I want to diet for the matric dance. The other reason was there was a time where we would go to trips and all of us would want to diet so that we could wear bikinis. (Pair 8)

However, almost all participants currently reported not dieting and that they did not diet for a long period:

But ha [giggles] I wouldn't survive like three days I'm done. (Pair 08)

[laughing] I don't know, but I can never stop eating junk. Maybe I would decrease a little but I would never stop eating it. (Pair 24)

## Discussion

Our findings illustrate the importance of qualitative research to better understand underlying social influences on behaviour. The study highlights how adolescent female best friends in an obesogenic environment of Soweto are engaged in shared food consumption and choices. Qualitative exploration revealed that it is common for best friends to share food and also share any healthy or unhealthy eating behaviour. This phenomenon was seen in multiple contexts, with most food sharing taking place around the purchase of fast foods at school and visits to the shopping mall. The pooling of money to purchase fast foods was an integral part of social support among friends and in some cases included economic planning. Eating together and sharing play an important role in the friendship, food sharing is part of the bidirectional influence friends have on each other^(^ ^30^ ^)^.

Our findings are consistent with results from Cape Town showing that most of the food purchased at school was classified as unhealthy (i.e. high in fat, added sugar and sodium, low in fibre and a low nutrient density)^(^ ^31^ ^)^. In our study, availability, quality and price of foods at school are important factors in food choice. The importance of money and availability of low-cost healthy options in choice of foods found in the present study is consistent with the literature^(^ ^18^ ^)^. In the shopping mall adolescents are less limited by availability and quality factors as compared with school. However, fast foods were still often bought and preferred. These results are consistent with other results from South Africa showing the popularity of fast food^(^ ^9^ ^)^. Our findings reinforce other research showing that healthier food choices are often less widely available^(^ ^12^ ^,^ ^32^ ^)^. Also, caregivers played an important role in determining food choices at school through providing the money for adolescents to purchase lunch at school.

There was a high prevalence of overweight and obesity rates in our study population and this prevalence exceeded proportions found in other similar aged South African female groups^(^ ^2^ ^,^ ^9^ ^)^. Dieting did not play a major role in the friendships, suggesting that the predominantly overweight girls are not contemplating action to reduce their weight. This possibly relates to the positive association of bigger body sizes with respect to happiness and beauty by South African females^(^ ^33^ ^)^. Hence, interventions to improve weight in this population should take this into consideration in their design.

Prior research on food consumption and friend influence has assumed the primary unit of data collection and analysis to be at the individual level. However, our findings in the South African context, similar to those of Mzicha *et al*.^(^ ^33^ ^)^, suggest the need to involve not only the targeted individual but also those close to the individual in health promotion activities. We strongly emphasize the importance of including the perspective of friends in future health interventions targeting obesity in South Africa. Shifts towards the Western diet and obesity starting at an earlier age^(^ ^10^ ^)^ confirm the need to intervene to reduce risk of obesity before adulthood. Globally, policy recommendations have also included calls for a reduction in portion size to address the obesity epidemic^(^ ^34^ ^)^. In cultures where friends commonly buy and share food together, more research is needed to adapt pricing and portion size strategies. The results related to sharing have important implications regarding price and portion size. Inconsistent with the findings of the study by French^(^ ^35^ ^)^, we observed that price does influence the food choices made at school. It was only in the school described as having ‘plenty of options’ where respondents reported buying fruits and the healthier school lunch. This was the only school where respondents reported pooling money to buy these meals. Food sharing may have positive influences by encouraging healthy food choices where large portions of healthy meals are sold at a low cost and shared among friends.

Future research should explore whether food sharing and pooling money also occur in other settings. In our study population, reasons given for these phenomena related to both social connection between friends as well as their economic situation. A comparison of findings in other contexts may complement the picture for the Sowetan setting by providing information as to the relative importance of both these factors in other contexts. The use of the duo-interviewing technique is recommended for future similar studies since this method allowed the researchers to observe the actual interaction among friends together while probing for in-depth understanding of choices. Because the two participants were already friends, it was relatively easy to build up a rapport and the participants were enthusiastic and reported enjoying participating in the research. As in other studies, it was less intimidating for the participants to speak since they were together with a friend^(^ ^28^ ^)^. Furthermore, high follow-up rates may be attributed to the fact that when pairs applied for participation they committed themselves not only to the researchers but also to each other. Unfortunately, it was not possible to document the response rate. All female students from grade 12 attending the target schools were eligible to participate in the study but it is unknown how many were present on the day students were informed. Furthermore, we only have information from those who came forward to participate. Therefore, it is not clear how those participants differ from other students. Additionally, it is important to note that in some cases the interview was dominated by one of the friends. In these cases, the interviewer specifically invited the less dominant participant to give her opinion. However, it is impossible to fully avoid this bias. It should also be noted that in the process of transcription it was sometimes difficult to distinguish the two friends in the transcript. As with any qualitative study the results are subject to the interpretation of the research team. Yet, individual bias was minimized through the rigorous approach to coding and the checking of those codes by four members of the research team.

## Conclusions

The qualitative duo-interview approach used has helped to identify critical information. Unique findings of the present study highlight that in Soweto a significant proportion of food is commonly decided upon and shared among friends. Also, joint decisions made were often unhealthy ones, influenced by availability in the school context and shaped by preference in the mall context. To tackle obesity, future research needs to explore the relationships between food sharing, portion size, pricing and the food choices friends make together in varying contexts. Future interventions need to recognize that a critical component may be to involve friends in the intervention design. Potential practical application of the current findings is the provision of desirable and price-competitive larger portions of healthy food. This may improve dietary intake of fruit and vegetables where a single portion is often shared among two or more friends.

## Footnotes

* C.G.N.V., MSc Health Sciences, Dutch national (native Dutch speaking); S.A.N., PhD Epidemiology, South African national (native English speaking); M.H.S., MSc Nutrition BSc Dietetics, South African national (native Northern Sesotho speaking); P.L.G., PhD Demography, UK national (native English speaking); M.J.W., PhD qualitative research methods, Dutch national (native Dutch speaking); C.M.D., MA Anthropology, PhD Nutrition, US national (native English speaking).

* At the time of the interview, a kota varied in price from R5 to R15, or $US 0·64 to $US 1·92.

* KFC and Wimpy are fast-food chains.

†
Lays is a brand of crisps, Pick ‘n Pay is a local chain of grocery stores.
